# No evidence to support the impact of migration background on treatment response rates and cancer survival: a retrospective matched-pair analysis in Germany

**DOI:** 10.1186/s12885-021-08141-8

**Published:** 2021-05-10

**Authors:** Roman Rüdiger, Franziska Geiser, Manuel Ritter, Peter Brossart, Mignon-Denise Keyver-Paik, Andree Faridi, Hartmut Vatter, Friedrich Bootz, Jennifer Landsberg, Jörg C. Kalff, Ulrich Herrlinger, Glen Kristiansen, Torsten Pietsch, Stefan Aretz, Daniel Thomas, Lukas Radbruch, Franz-Josef Kramer, Christian P. Strassburg, Maria Gonzalez-Carmona, Dirk Skowasch, Markus Essler, Matthias Schmid, Jennifer Nadal, Nicole Ernstmann, Amit Sharma, Benjamin Funke, Ingo G. H. Schmidt-Wolf

**Affiliations:** 1 Department of Integrated Oncology, CIO Bonn, Center for Integrated Oncology ABCD, University Hospital Bonn, Venusberg-Campus 1, 53127 Bonn, Germany; 2 Institute of Psychosomatic Medicine and Psychotherapy, University Hospital Bonn, Bonn, Germany; 3 Department of Urology, University Hospital Bonn, Bonn, Germany; 4 Department of Internal Medicine III, University Hospital Bonn, Bonn, Germany; 5 Department of Senology and certified Breast Center, University Hospital Bonn, Bonn, Germany; 6 Department of Neurosurgery, University Hospital Bonn, Bonn, Germany; 7 Department of Otorhinolaryngology, University Hospital Bonn, Bonn, Germany; 8 Department of Dermatology and Allergy, University Hospital Bonn, Bonn, Germany; 9 Department of Surgery, University Hospital Bonn, Bonn, Germany; 10 Department of Neurology, University Hospital Bonn, Bonn, Germany; 11 Institute of Pathology, University Hospital Bonn, Bonn, Germany; 12 Department of Neuropathology, University Hospital Bonn, Bonn, Germany; 13 Institute of Human Genetics, University Hospital Bonn, Bonn, Germany; 14 Department of Radiology, University Hospital Bonn, Bonn, Germany; 15 Department of Palliative Medicine, University Hospital Bonn, Bonn, Germany; 16 Department of Oral and Maxillofacial Plastic Surgery, University Hospital Bonn, Bonn, Germany; 17 Department of Internal Medicine I, University Hospital Bonn, Bonn, Germany; 18 Department of Internal Medicine II, University Hospital Bonn, Bonn, Germany; 19 Department of Nuclear Medicine, University Hospital Bonn, Bonn, Germany; 20 Institute for Medical Biometry, Computer Science and Epidemiology, University Hospital Bonn, Bonn, Germany; 21 Center for Health Communication and Health Services Research, Department of Psychosomatic Medicine and Psychotherapy, University Hospital Bonn, Bonn, Germany

**Keywords:** Cancer, Migration background, Matched-pair analysis, Response rate, Survival, Socioeconomic status, Comprehensive cancer center, Germany

## Abstract

**Background:**

Immigration has taken the central stage in world politics, especially in the developed countries like Germany, where the continuous flow of immigrants has been well documented since 1960s. Strikingly, emerging data suggest that migrant patients have a poorer response to the treatment and lower survival rates in their new host country, raising concerns about health disparities. Herein, we present our investigation on the treatment response rate and cancer survival in German patients with and without an immigrant background that were treated at our comprehensive cancer center in Germany.

**Methods:**

Initially, we considered 8162 cancer patients treated at the Center for Integrated Oncology (CIO), University Hospital Bonn, Germany (April 2002–December 2015) for matched-pair analysis. Subsequently, the German patients with a migration background and those from the native German population were manually identified and catalogued using a highly specific name-based algorithm. The clinical parameters such as demographic characteristics, tumor characteristics, defined staging criteria, and primary therapy were further adjusted. Using these stringent criteria, a total of 422 patients (*n* = 211, Germans with migration background; n = 211, native German population) were screened to compare for the treatment response and survival rates (i.e., 5-year overall survival, progression-free survival, and time to progression).

**Results:**

Compared to the cohort with migration background, the cohort without migration background was slightly older (54.9 vs. 57.9 years) while having the same sex distribution (54.5% vs. 55.0% female) and longer follow-up time (36.9 vs. 42.6 months). We did not find significant differences in cancer survival (5-year overall survival, *P* = 0.771) and the response rates (Overall Remission Rate; McNemar’s test, *P* = 0.346) between both collectives.

**Conclusion:**

Contrary to prior reports, we found no significant differences in cancer survival between German patients with immigrant background and native German patients. Nevertheless, the advanced treatment protocols implemented at our comprehensive cancer center may possibly account for the low variance in outcome. To conduct similar studies with a broader perspective, we propose that certain risk factors (country-of-origin-specific infections, dietary habits, epigenetics for chronic diseases etc.) should be considered, specially in the future studies that will recruit new arrivals from the 2015 German refugee crisis.

## Background

Immigration has become a global issue, and there has been a plethora of research published on the socioeconomic and demographic determinants of diseases associated with the health of immigrants. In particular, the migration-related diversity can often lead to inequalities in essential domains of life, as different health requirements and inequalities may rely on the divergent lifestyle factors and the average socioeconomic status [1]. In context to cancer, there have been few studies that have examined the association between ethnic background and cancer, primarily in Europe. For instance, Hemminki et al. investigated the prostate cancer incidence and survival among immigrants in Sweden and showed that non-European male immigrants (mainly from the Middle East, Asia, and Chile) harbor the lowest risk with the most favorable survival [2]. Likewise, Mousavi et al. assessed the possible ethnic differences in breast cancer risk and survival among immigrants in Sweden but did not find any substantial evidence [3]. A study examining the survival of first-generation non-Western immigrants with stomach cancer residing in the Northeastern Netherlands also reported about inconclusive results [4].

Over the years, Germany has exponentially become a popular destination for immigrants from Europe, and also from other continents. According to the recent report (year 2018) from the Federal Office for Migration and Refugees, nearly a quarter (20.8 million) of the 81.6 million people living in Germany had a migration background and around 10.9 million (52.4%) have already received the German citizenship. The overall share of German citizens with a migrant background in the total population amounts to 13.4% [5]. Interestingly, one study analyzed the cross-national prospective of cancer incidences among ethnic Germans who migrated from the former Soviet Union to Germany (also named as resettlers) and those residing in Russia, and concluded that the incidence among migrant populations often remains the same between the population of origin and the new host community [6, 7]. Given that Germany is home to ~ 4 million people with Turkish roots, Spallek et al. found in a similar cross-national study that cancer of the respiratory organs is diagnosed less frequently in Turkish men in older birth cohorts, whereas it is more frequent in younger birth cohorts [8]. In a very similar context, Spix et al. analyzed childhood cancer survival among children of Turkish descent in the German Cancer Childhood Registry and reported about a small group of Turkish children with lymphoid leukemia with significantly lower survival [9].

We also recently discussed cancer survival and response rates between German and foreign patients and raised some important concerns, such as undermining the impact of nationality on survival and the rational goal of creating a fair health care system for all the patients [10]. Now, as an extension of our previous study, herein, we compared the German cancer patients with a migration background with patients from the native German population. We specifically investigated the aforementioned 52.4% of German immigrants, and new immigrants from 2015 refugee crisis tend not to be included in this study.

To our knowledge, this is the first study that has examined the effect of ethnicity in such a large variety of cancers (17 tumor entities) in any immigrant population in Germany.

## Methods

### Patients

Initially, we considered 8162 cancer patients treated at the Center for Integrated Oncology (CIO), University Hospital Bonn, Germany (April 2002–December 2015) for matched-pair analysis. Subsequently, the German patients with a migration background and those from the native German population were manually identified and catalogued using an algorithm which has been proven to have a very good performance (sensitivity and specificity ≥0.975). This algorithm has already been used by Spelleck et al. to exclusively differentiate Turkish names from Greek and Arabic ones, and to further differentiate such names from German counterparts [11]. Since we catalogued only distinctive Arabic names within the German population, the algorithm’s quality remains integral to our analysis. Briefly, the majority of patients with a migration background were identified by their full name, i.e., only if their first and last name(s) were clearly foreign and holds German citizenship, as per their medical record.

We also included a smaller number of cases where one part of the name was certainly foreign and the other part was a doublet, suggesting that it could be either German or foreign. In order to exclude misidentifications, we additionally considered information like patients’ native language and/or names of relatives etc. Using these strict criteria, we finally screened 422 patients (*n* = 211, migration background; n = 211, native German population).

The small number of patients with migrant background (2.6%) that contrasts with the significantly higher proportion of naturalized immigrants in Germany (13.4%) it arises due to enrolling patients with a unique foreign name.

### Matched-pair analysis

After classifying the patients with a migration background and/or native German population, we applied the following pairing criteria: age difference ± 10 years, sex, diagnosis according to ICD-10 (grouped by 3-digit codes) and ICD-O-3 (grouped by 5-digit codes) or equivalent, disease status (primary case vs. recurrence) and tumor stage (UICC stage and grading for solid tumors, Durie and Salmon Staging System for multiple myeloma, Ann Arbor score for lymphomas, Binet status for CLL and WHO classification for tumors of the CNS). In addition, the following morphologic features were included to improve the matching process: the Gleason score for prostate cancer patients, the FAB classification for AML, Breslow’s depth, and Clark’s level for malignant melanoma. The estrogen, progesterone and erbB-2 receptor status of breast cancer patients were also used to find a suitable match. The matching partners with convergent treatment were found for 211 of 226 patients (93.4%) in the primary therapy, while the remaining 15 patients were excluded from further analysis. The slight differences in chemotherapeutic agents, immunotherapy or hormone therapy regimens, or in the use of adjunctive therapies were observed, which were tolerated as they affected only a small number of patients and were consistent with the main treatment procedure. As we accepted the age difference of 10 years, the possible significant differences between the two cohorts of patients could be excluded for all matching parameters, except the age of patients. The main patient characteristics are shown in Table 1, notably, the cohort with migration background was on average a few years younger than the other cohort (54.9 vs. 57.9 years, respectively). We observed that the majority of subjects in our analysis were diagnosed within the interval of 2 years, therefore, we also incorporated this information as an additional matching criterion (Table 2). The frequencies of tumor-specific matching criteria are shown in Table 3. To mention, we analyzed 17 different tumor entities, therefore, we performed statistical analysis only for those variables that had a sufficient number of comparable cases.

**Table 1:** Patient characteristics

		Patients				
		Native Germans		Germans with migrant background		*p*-value
		n	%	n	%	
Total		211	100.0	211	100.0	
Sex	Female	116	55.0	115	54.5	0.796^a^
	Male	95	45.0	96	45.5	
Age	< 31	9	4.3	17	8.1	0.174^b^
	31–60	107	50.7	116	55	
	61–80	90	42.7	72	34.1	
	> 80	5	2.4	6	2.8	
Tumor entities	Head and neck cancer	15	7.1	15	7.1	N.d.^c^
	Gastrointestinal cancer	20	9.5	20	9.5	
	Lung cancer	5	2.4	5	2.4	
	Skin cancer	19	9.0	19	9.0	
	Gynecologic cancer	64	30.3	64	30.3	
	Urological cancer	35	16.6	35	16.6	
	CNS cancer	19	9.0	19	9.0	
	Thyroid cancer	12	5.7	12	5.7	
	Tumors of the hematopoietic and lymphoid tissues	22	10.4	22	10.4	
Treatment	None	3	1.4	3	1.4	N.d.
	Chemotherapy	20	9.5	20	9.5	
	Radiotherapy	2	0.9	2	0.9	
	Resection	70	33.2	70	33.2	
	Radiotherapy + resection	32	15.2	32	15.2	
	Chemoradiotherapy	10	4.7	10	4.7	
	Chemotherapy + stem cell therapy	6	2.8	6	2.8	
	Chemotherapy + resection	27	12.8	27	12.8	
	Chemoradiotherapy + resection	41	19.4	41	19.4	

^a^ McNemar’s test

^b^ Bowker’s test

^c^ Not done because of small sample size

**Table 2:** Differences in year of diagnosis

Mean		0.024
95% Confidence Interval	Lower Limit	−0.351
	Upper Limit	0.399
Standard Deviation		2.763
Minimum		−10
Maximum		9

**Table 3:** Frequencies of tumor-specific characteristics

			Patients			
			Native Germans		Germans with migrant background	
			n	%	n	%
Solid tumors	UICC classification	Total	168		167	
		Stage 0	1	0.6	1	0.6
		Stage 1	80	47.6	78	46.7
		Stage 2	35	20.8	35	21
		Stage 3	24	14.3	26	15.6
		Stage 4	28	16.7	27	16.2
	Histological grading	Total	112		117	
		G1	12	10.7	13	11.1
		G2	57	50.9	57	48.7
		G3	43	38.4	47	40.2
Lymphomas	Ann Arbor score	Total	8		8	
		Stage 1	1	12.5	1	12.5
		Stage 2	2	25.0	2	25.0
		Stage 3	1	12.5	1	12.5
		Stage 4	4	50.0	4	50.0
Haemotolgic malignancies	FAB classification	Total	19		19	
		Grade I	1	5.3	1	5.3
		Grade II	2	10.5	2	10.5
		Grade III	3	15.8	3	15.8
		Grade IV	13	68.4	13	68.4
Prostatic cancer	Gleason score	Total	9		10	
		6	3	33.3	3	30.0
		7	5	55.6	4	40.0
		8	0	0	1	10.0
		9	1	11.1	2	20.0
Malignant melanoma	Breslow’s depth	Total	17		17	
		Stage 1	6	35.3	5	29.4
		Stage 2	3	17.6	3	17.6
		Stage 3	3	17.6	4	23.5
		Stage 4	2	11.8	2	11.8
		Stage 5	3	17.6	3	17.6

### Statistical analysis

SAS statistical analysis software (version 9.4 by SAS Institute Inc., Cary, NC, USA for Windows) was used for the statistical analyses. Nominal and ordinal variables were assessed using McNemar’s test for 2 × 2 tables. Bowker’s test was used for tables larger than 2 × 2. All tests were two-sided and *p* < 0.05 was preset as the cutoff for significance. Since there is no adjustment for multiple tests, *p*-values should be interpreted as exploratory only.

We estimated hazard ratios (HRs) with 95% confidence intervals (95% CI) for the primary endpoints of this study, 5-year overall survival, progression-free survival, and time to progression, using a conditional logistic regression model. Besides the regression models, the outcomes were also represented with Kaplan-Meier curves. The overall survival (point of diagnosis till death) was limited to a 5-year period (5-y OS) as only a small number of events occurred at a later point in time. Time to progression (TTP) was defined as the time between the date of diagnosis and the date of disease progression or death related to cancer. Progression-free survival (PFS) was defined as the time starting with the date of diagnosis leading up to the date of disease progression or death from any cause.

Response rate was evaluated according to Response Evaluation Criteria in Solid Tumors (RECIST) and was classified as complete remission (CR), partial remission (PR), stable disease (SD) and progressive disease (PD).

## Results

### Patients’ characteristics

#### Patients from native German cohort

The mean age of the native German patient cohort was 57.9 years (*n* = 211, range 22–88 years). Of these, three additional subgroups were defined (*n* = 157, range 41–70 years; *n* = 18, range < 41 years; *n* = 36, range > 70 years), accounting for 74.4, 8.5 and 17.1%, respectively. We also characterized the cohort according to sex basis (males: *n* = 95, 45%; females: *n* = 116, 55%), and included mainly the patients (*n* = 183, 86.7%) with an evaluable insurance status (National health insurance: *n* = 110, 60%; Private health insurance: *n* = 45, 24.6%; Self-payers: *n* = 28, 15.3%). The mean follow-up time for the entire patient group was 42.6 months (range, 0.7–156.3 months).

#### Patients with migration background

The mean age of the migration patient cohort was 54.9 years (*n* = 211, range 21–92 years). Here again, three additional subgroups were defined (*n* = 134, range 41–70 years; *n* = 42, range < 41 years; *n* = 35, range > 70 years), accounting for 63.5, 19.9, and 16.6%, respectively. This cohort was also characterized according to the sex basis (males: *n* = 96, 45.5%; females: *n* = 115, 54.5%), and included mostly the patients (*n* = 172, 81.5%) with a valid insurance status (National health insurance: *n* = 141, 82%; Private health insurance: *n* = 13, 7.6%; Self-payers: *n* = 18, 10.5%). In this cohort, the mean follow-up time for the entire patient group was 36.9 months (range 0.2–151 months).

### Matched-pair characteristics

Most patients were included with a primary tumor (*n* = 207, 98.1%) and few with a recurrence (*n* = 4, 1.9%). The matched-pair cases were mainly diagnosed with a solid tumor (*n* = 189, 89.6%) and few (*n* = 22, 10.4%) having hematological malignancies. In the matched-pair analyses, breast cancer emerged as the largest tumor entity (*n* = 60 pairs, 28.4%), followed by urological cancer (*n* = 35 pairs, 16.6%), hematopoietic and lymphoid tissue tumors (n = 22 pairs, 10.4%), gastrointestinal tract cancer (n = 20 pairs, 9.5%), CNS tumors (*n* = 19 pairs, 9%), and head and neck cancer (*n* = 15 pairs, 7.1%).

### Response to treatment

When the native German cohort was compared with the migrant cohort, using RECIST criteria, only minor differences in response to treatment were observed (Table 4). As the therapy/treatment data were available for the majority of patients (native German cohort: *n* = 209, 99%; migration cohort: *n* = 211, 100%), we were able to evaluate additional clinical parameters such as: Complete Remission/CR (native German cohort: *n* = 150, 71.8%; migration cohort: *n* = 154, 73%), Partial remission/PR (native German cohort: n = 20, 9.6%; migration cohort: n = 20, 9.5%), Stable disease/SD (native German cohort: n = 15, 7.2%; migration cohort: *n* = 10, 4.7%), and a Progressive disease/PD (native German cohort: *n* = 24, 11.5%; migration cohort: *n* = 27, 12.8%). Importantly, all of these clinical parameters appeared to be statistically non-significant (Bowker’s test, *P* = 0.487). Importantly, when the overall response rate (ORR) that combines CR/PR and SD/PD was checked, the differences between the two groups remained minimal. For instance, the values obtained were: CR/PR (native German cohort, *n* = 170, (81.3%); migration cohort, *n* = 174, 82.5%) and for SD/PD (native German cohort, *n* = 39, (18.7%); migration cohort, *n* = 37, 17.5%), (McNemar’s test, *P* = 0.346).

**Table 4:** Response to treatment

		Patients				
		Native Germans		Germans with migrant background		*p*-value
		n	%	n	%	
RECIST	CR	150	71.8	154	73	0.487^a^
	PR	20	9.6	20	9.5	
	SD	15	7.2	10	4.7	
	PD	24	11.5	27	12.8	
ORR	CR + PR	170	81.3	174	82.5	0.832^b^
	SD + PD	39	18.7	37	17.5	

^a^ Bowker’s test

^b^ McNemar’s test

### Survival analysis

**Fig. 1:**
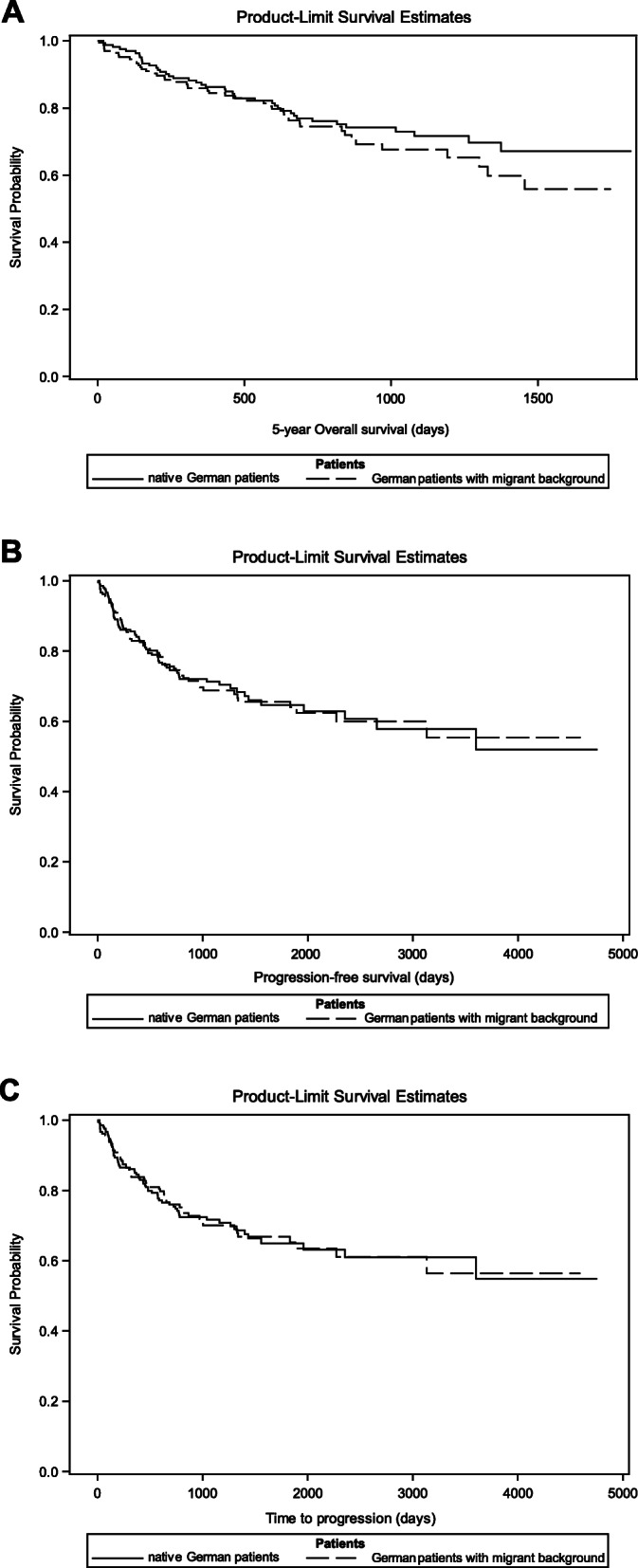
Kaplan-Meier curves including all entities. **a** 5-year overall survival (5-y OS) of all patients with and without migration background (*n* = 422). The mean OS was 34.8 months for the native German cohort (*n* = 211) and 30.8 months for the migration cohort (*n* = 211) (conditional logistic regression model; HR 0.951, 95% CI [0.670;1.349]; *p* = 0.771). **b** Progression-free survival (PFS) of all patients with and without a migration background (*n* = 422). The mean PFS was 23.5 months for the native German cohort (*n* = 211) and 22.5 months for the patients with migration cohort (*n* = 211) (HR 0.957, 95% CI [0.719;1.275]; *p* = 0.766). **c** Time to progression (TTP) of all patients with and without a migration background (*n* = 422). The mean TTP was 24.1 months for the native German cohort (*n* = 211) and 22.8 months for the migration cohort (*n* = 211) (conditional logistic regression model; HR 0.947, 95% CI [0.709;1.236]; *p* = 0.709)

A conditional logistic regression model was used to compare the survival of both patient groups (native Germans versus migration) (Table 5), the graphical illustration was supplemented with Kaplan Meier curves (Fig. 1).

**Table 5:** Conditional logistic regression model

Outcome	HR	CI	*p*-value
5-year Overall survival	0.951	[0.670; 1.349]	0.771
Progression-free survival	0.957	[0.719; 1.275]	0.7660
Time to progression	0.947	[0.709; 1.263]	0.7094

#### 5-year overall survival (5-y OS)

The mean 5-y OS was found to be 34.8 months for the native German cohort (*n* = 211) and 30.8 months for the migration cohort (*n* = 211) (HR 0.951, 95% CI [0.670,1.349]; *p* = 0.771). There was also a considerable number of patients in both cohorts who had died during the course of study (native Germans: *n* = 42, 19.9%; migration: *n* = 46, 21.8%).

#### Progression-free survival (PFS)

The mean PFS was found to be 23.5 months for the native German cohort (*n* = 211) and 22.5 months for the migration cohort (n = 211) (HR 0.957, 95% CI [0.719;1.275]; *p* = 0.766). Also, there was a considerable number of patients in both cohorts who had experienced disease progression or died due to varying causes (native Germans: *n* = 65, 30.8%; migration: *n* = 63, 29.9%).

#### Time to progression (TTP)

The mean TTP was found to be 24.1 months for the native German cohort (n = 211) and 22.8 months for the migration cohort (*n* = 211) (HR 0.947, 95% CI [0.709;1.236]; *p* = 0.709). There was also a substantial number of patients in both cohorts who had experienced disease progression or died of cancer (native Germans: n = 63, 29.9%; migration: *n* = 60, 28.4%).

## Discussion

There has been growing interest in studying the impact of immigration on cancer incidence, and it has been suggested that cancer rates across migrant descendants may be approaching the same level as of home/host country [12, 13]. Whereas most studies have focused on socioeconomic and demographic factors as the primary cause of variance in cancer survival data, few have also discussed about the health behaviors and perceptions of immigrants that differ from native-born cancer survivors [14]. The main objective of our current study was to investigate the cancer survival rate in German patients with and without migration background, primarily, treated in our comprehensive cancer center at the University Hospital Bonn, Germany. It is important to clarify that our study focuses mainly on the cancer mortality and differs from other ongoing approaches that examine the incidence of risk patterns influencing the cancer development. In our analysis, we found no evidence that the migration background of cancer patients significantly affects the response rates and/or survival. The Kaplan-Meier curves for the overall survival from the cumulative patient’s data display a specific trend i.e., the immigrants show consistently lower but non-significant survival rates than the native German patients. Although both curves subsequently converged in later stages but they contained very few notable events. To mention, the ability to further differentiate subgroups of migrants (1st/2nd/later generation) to clarify differences in the overall survival curve at very early stages remains a challenge in registry-based studies. Moreover, with the name-based approach, it is also not possible to distinguish between the subsequent generations [15].

Hemminki et al. have reported that certain cancers such as liver, nasopharyngeal, esophageal, gastric, and cervical cancers are associated with microbial infections, nutritional imbalances and toxins [16]. This is indeed an important concern that should be addressed in future studies dealing with new arrivals in Germany during the 2015 refugee crisis, but in our current study, the enrolled immigrants arrived several years ago and/or are at least the second-generation of migrants in Germany. As we found no significant differences in cancer survival between German patients with migrant background and the native German population, our data strongly pointed towards the adaptation of immigrants to the country-specific cancer survival patterns. In context to socioeconomic status, we reviewed the information about the different insurances (national health insurance, private health insurance, self-pay policy) that our enrolled patients have used and found that the migrant patients used significantly less private health insurance policies (7.6%) compared to the native Germans (24.6%) (Bowker’s test, *P* < 0.05). This also supports the general assumption that the patients with an immigrant background tend to have a lower socioeconomic status. As suggested by Spallek et al., that morbidity and mortality risks of migrants can differ considerably from those of populations in the host countries, therefore, it is necessary to document a variety of different factors that might distinguish the second or third generation from the parent generation of migrants. In this context, we propose that certain risk factors (country-of-origin-specific infections, dietary habits, alcohol consumption, smoking behavior, obesity/body weight index and epigenetics for chronic diseases) should be considered, especially in the future studies that will recruit new arrivals from the 2015 German refugee crisis.

It is noteworthy to mention some limitations of our study that are: a) the manual name-based enrollment of migrant patients: as in rare cases first name/surname doublet may lead to the misidentification, especially when the background information is unavailable. In addition, such criteria cannot define the country of origin among immigrant subgroups. Since we included only immigrants with German citizenship (not new arrivals from the 2015 refugee crisis), the variance in our data due to such rare cases is at least slightly mitigated; b) the lack of info about the confounders: e.g., the disparities in degree of comorbidity could have improved the analysis as stable matching criterion; c) we included 17 different tumor entities with most having few matches, which in turn results into data heterogeneity with a limited statistical power.

## Conclusion

To our knowledge, this is the first study to examine the cancer survival in German patients with and without migration background using a wide range of different tumor entities. Our major conclusions are: a) we did not find any significant differences in cancer survival between patients of migration background and of native German population. Thus, our study strongly suggests about the equality of cancer survival in patients from different ethnic groups being treated in a comprehensive cancer center in Germany; b) a name-based approach to identify immigrants in a heterogeneous population may have limited efficacy; c) in future studies, more accurate information on the immigrant’s origin and region-specific risk factors may help better to differentiate the subgroups of migrants.

## Data Availability

The datasets used and analyzed during the current study are available from the corresponding author on request.

## Abbreviations

CIO: Center for Integrated Oncology
CR: Complete remission
ORR: Overall remission rate
OS: Overall survival
PD: Progressive disease
PFS: Progression-free survival
PR: Partial remission
RECIST criteria: Response evaluation criteria in solid tumors
SD: Stable disease
TTP: Time to progression
5-y OS: 5-year overall survival
